# Genome-Wide Linkage Mapping for Mixograph Properties in Common Wheat

**DOI:** 10.3390/plants15071016

**Published:** 2026-03-26

**Authors:** Qiqi Zhang, Fangfang Liu, Wenxin Cao, Yao Li, Yuxia Lv, Heng Zhou, Xin Du, Yingxiu Wan, Chuanxi Ma

**Affiliations:** 1 Key Laboratory of Wheat Biology and Genetic Improvement on Southern Yellow & Huai River Valley, Ministry of Agriculture, College of Agronomy, Anhui Agricultural University, Hefei 230036, China; zhqq1982@126.com (Q.Z.);; 2 Crop Research Institute, Anhui Academy of Agricultural Sciences, Bio-Breeding Laboratory of Anhui Province, Anhui Key Laboratory of Crop Quality Improvement, Hefei 230031, China

**Keywords:** wheat, Mixograph properties, QTL mapping, KASP marker, marker-assisted selection

## Abstract

Mixograph properties represent important quantitative traits that are controlled by multiple genes and influenced by environmental factors. In this study, we conducted quantitative trait locus (QTL) mapping for key Mixograph paraments using a recombinant inbred line (RIL) population derived from a cross between Yangxiaomai and Zhongyou 9507. Based on a high-density genetic map, six stable QTLs were identified on chromosomes 1A, 1B, and 1D across four environments, with individual phenotypic variation explained, ranging from 2.26 to 28.70%. Among these, *QTh.ahau-1A*, *QMt*/*QPa.ahau-1B*, and *QTw.ahau-1D.1* are potentially novel loci. Furthermore, four functional Kompetitive Allele-Specific PCR (KASP) markers were developed based on tightly linked SNPs and validated in 110 advanced breeding lines, confirming their significant association with the target traits and utility for marker-assisted selection (MAS). Additionally, six candidate genes were predicted, which encoded proteins such as a hydroxyproline-rich glycoprotein, a CCCH-type zinc finger protein, protease, kinase, a phosphoglucan water dikinase, and a TRP-like family protein. Collectively, these findings provide valuable genetic loci, functional molecular markers, and candidate gene resources for improving wheat processing quality through MAS-based breeding.

## 1. Introduction

Dough rheological properties are key indices for evaluating wheat flour quality, as they comprehensively reflect the mixing tolerance and viscoelasticity of dough, thereby directly influencing the quality of end-use wheat products [1,2]. Internationally established instruments for measuring these properties include the Farinograph, Extensograph, Alveograph, Mixograph, and Mixolab. Among them, the Farinograph, Extensograph, and Alveograph typically require 300–350 g of flour per test and involve prolonged assay times, which limits their applicability for high-throughput screening and early-generation selection in breeding programs. Mixolab is a novel device capable of simultaneously determining both the Farinograph properties and starch viscosity characteristics of flour. However, its standard method still requires 50 g of flour and a testing time of approximately 45 min [3]. In contrast, the Mixograph, developed in the 1930s, provides notable advantages, including low sample consumption and rapid testing [4]. The parameters derived from Mixograph curves, such as mixing time, peak height, peak width, peak area, 8 min tail height, and 8 min tail width, provide key rheological data. Collectively, they offer a comprehensive profile of dough properties. Studies have demonstrated strong correlations between Mixograph parameters and those obtained from the Farinograph, Extensograph, and actual baking performance [5,6]. Consequently, the Mixograph has been extensively adopted in wheat breeding programs and quality assessment worldwide.

Mixograph properties are typical quantitative traits controlled by multiple genes and influenced by environmental factors. Their genetic dissection mainly relies on quantitative trait locus (QTL) mapping and genome-wide association studies (GWASs). Early QTL mapping efforts, primarily utilizing genetic populations such as recombinant inbred lines (RILs) or doubled haploids (DHs), led to the identification of multiple major loci parameters, such as controlling peak height and width on chromosomes 1A, 1B, 3A, and 4D [7,8]. Subsequent studies confirmed important loci on chromosomes 1B and 1D [9,10], while further work reported over a hundred loci associated with Farinograph and Mixograph parameters in DH populations [11]. With the application of high-density genetic maps, the precision of QTL mapping has been significantly improved. For instance, stable QTLs have been identified across environments, with the Glu-D1 locus explaining 27.1–36.7% of the phenotypic variation for midline peak height, representing a core major locus [12]. The importance of other chromosomal regions, such as Glu-A1, Glu-D1, 3B, and 6A, has also been established [10,13]. In recent years, the application of GWAS technology has greatly expanded the scope of identifying loci associated with Mixograph parameters. Although high-throughput SNP studies have revealed the widespread distribution and pleiotropy of loci controlling dough rheology, along with numerous population-specific markers and QTLs, key limitations persist. These include insufficient marker density, unstable QTL effects across environments, excessively large confidence intervals, and a lack of identified causal genes. Thus, the genetic architecture underlying Mixograph parameters requires further investigation.

In this study, an RIL population derived from a cross between Yangxiaomai and Zhongyou 9507 was used. The wheat variety Zhongyou 9507 demonstrated superior dough rheological properties and excellent processing quality. The landrace variety, Yangxiaomai, typically exhibits moderate end-use quality. The genetic basis of Mixograph parameters was dissected using a high-density 55 K genetic map, related QTLs were identified, and corresponding high-throughput KASP markers were developed, with the aim of providing practical molecular tools for marker-assisted selection (MAS) in wheat breeding.

## 2. Results

### 2.1. Phenotypic Variation in Mixograph Parameters

**Figure 1:**
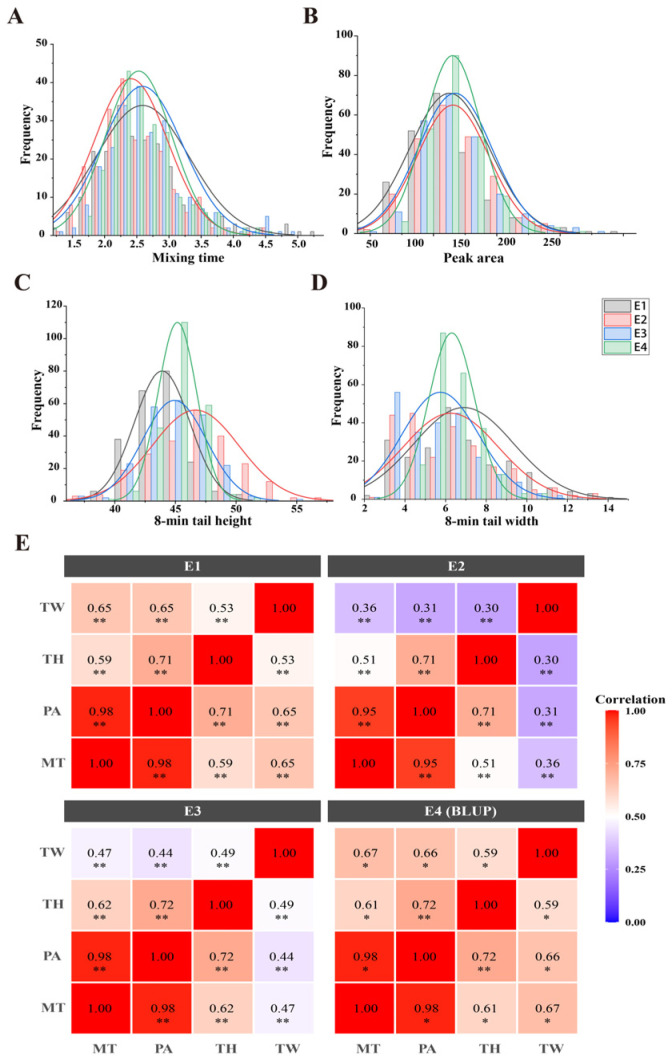
Frequency distribution and correlation analysis of Mixograph parameters in RIL population. (**A**) Frequency distribution of mixing time in four enviroments. (**B**) Frequency distribution of peak area in four enviroments. (**C**) Frequency distribution of 8-min tail height in four enviroments. (**D**) Frequency distribution of 8-min tail width in four enviroments. (**E**) Correlation analysis of Mixograph parameters in four enviroments. MT: mixing time; PA: peak area; TH: 8 min tail height;8 min tail width. E1, E2, E3, and E4 indicate Suixi 2021, Suixi 2022, Hefei 2022, and the best linear unbiased prediction (BLUP), respectively. * *p* < 0.05, ** *p* < 0.01.

Phenotypic data of Mixograph parameters for parents and RIL lines planted during 2020–2022 were statistically analyzed. As shown in Table 1, Zhongyou 9507 exhibited higher values than Yangxiaomai in mixing time (MT), peak area (PA), 8 min tail height (TH), and 8 min tail width (TW) in E1, E2, E3, and E4 (BLUP). In all environments, the above parameters of the RIL population showed a roughly normal distribution (Figure 1A–D), while some lines exhibited transgressive segregation. Across environments, the coefficient of variation (CV) for all traits ranged from 0.04 to 0.39, with the highest value recorded for TW. The correlation analysis revealed extremely significant positive correlations among all traits across different environments (Figure 1E). The correlation coefficient between MT and PA was the highest (0.95–0.98).

The ANOVA results showed significant differences (*p* < 0.01) in MT, PA, TH, and TW among genotypes, environments, and genotype–environment interactions (Table 2). The *H*^2^ of mixing time, peak area, 8 min tail height, and 8 min tail width were 0.90, 0.87, 0.77, and 0.74, respectively, indicating that these traits are mainly influenced by genetic factors (Table 2).

### 2.2. QTL Mapping Results

Six stable major QTLs associated with Mixograph properties were identified in the RIL population across four environments (E1–E4), distributed on chromosomes 1A, 1B, and 1D (Table 3). Individual QTLs explained 2.26–28.70% of the phenotypic variation, with an average of 10.15% (Table S1, Figure S1).

Two major QTLs were detected on chromosome 1A. *QMt.ahau-1A*, associated with mixing time, was located between SNP markers *AX-110911310* and *AX-108914408*, with a confidence interval of 109.5–110.5 cM, corresponding to a physical position of 513.62–514.56 Mb on the Chinese Spring reference genome (IWGSC v1.0). It was detected in both E2 and E3 environments, explaining 13.31–17.36% of the phenotypic variation with additive effects of 0.22–0.24, and its favorable allele was derived from Zhongyou 9507. *QTh.ahau-1A*, associated with 8 min tail height, was located between SNP markers *AX-109055961* and *AX-111496677*, corresponding to a physical position of 504.82–508.05 Mb. It was detected in both E2 and E4 environments, explaining 16.63–21.13% of the phenotypic variation with additive effects of −1.15 to −1.06, and its favorable allele was derived from Yangxiaomai.

A major pleiotropic QTL controlling both MT and PA was detected on chromosome 1B. *QMt.ahau-1B*, associated with mixing time, was located between SNP markers *AX-111503025* and *AX-110519906* (168.5–172.0 cM; physical position 551.72–552.48 Mb). It was detected in E1, E2, and E4, explaining 19.49–28.70% phenotypic variation with additive effects of 0.30–0.45, and its favorable allele derived from Zhongyou9507. *QPa.ahau-1B*, at the same position, explained 19.52–23.92% of the phenotypic variation with additive effects of 11.52–16.85, with the favorable allele also derived from Zhongyou 9507.

Two major QTLs for TW were detected on chromosome 1D. *QTw.ahau-1D.1* was located between SNP markers *AX-109827648* and *AX-110069135* (166.5–167.5 cM; physical position 374.05–375.94 Mb). It was detected in E1 and E2, explaining 7.73–12.04% of the phenotypic variation, with additive effects of −0.78 to −0.35; its favorable allele was derived from Yangxiaomai. *QTw.ahau-1D.2* was located between SNP markers *AX-109079356* and *AX-111804778* (198.5–199.5 cM; physical position 410.07–413.66 Mb). It was detected in E1, E3, and E4, explaining 4.09–17.21% of the phenotypic variation with additive effects of 0.83–0.85; its favorable allele was derived from Zhongyou 9507.

### 2.3. Effect Analysis of Stable Loci

**Figure 2:**
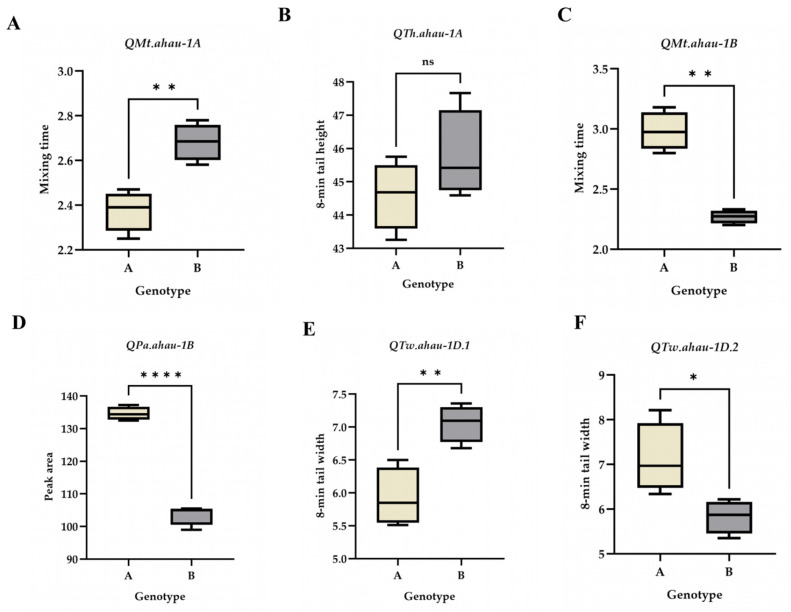
Effect analysis of stable loci on Mixograph parameters of RIL population. (**A**) Effect analysis of *QMt.ahau-1A*. (**B**) Effect analysis of *QTh.ahau-1A*. (**C**) Effect analysis of *QMt.ahau-1A*. (**D**) Effect analysis of *QPa.ahau-1B*. (**E**) Effect analysis of *QTw.ahau-1D.1*. (**F**) Effect analysis of *QTw.ahau-1D.2*. ns: not significant, * *p* < 0.05, ** *p* < 0.01, **** *p* < 0.0001.

The favorable alleles of the identified QTLs significantly increased the values of their corresponding Mixograph traits (Figure 2). Specifically, lines carrying the favorable allele of *QMt.ahau-1A* showed an 8.50–14.03% increase in MT (*p* < 0.01); those carrying the favorable allele of *QMt.ahau-1B* exhibited a greater increase of 27.27–40.71% (*p* < 0.01). For TW, the favorable alleles of *QTw.ahau-1D.1* and *QTw.ahau-1D.2* increased the trait by 9.44–24.73% (*p* < 0.05) and 15.24–31.99% (*p* < 0.05), respectively. The favorable allele of *QPa.ahau-1B* led to an increase 26.20–38.51% in PA (*p* < 0.001). In contrast, the favorable allele of *QTh.ahau-1A* only increased TH by 1.30–3.17%, which was not statistically significant.

### 2.4. Development of KASP Markers and Validation in Advanced Lines 

To facilitate the marker-assisted selection of the identified QTLs, SNP markers tightly linked to *QMt.ahau-1A*, *QMt.ahau-1B*, and *QTw.ahau-1D.1* were converted into KASP markers. Four KASP markers (*QMt-1A-KASP*, *QMt-1B-KASP*, *QTw-1D.1-KASP-1*, and *QTw-1D.1-KASP-2*) were successfully developed (Table 4). The primers were designed using the online tool Polymarker, with allele-specific forward primers labeled with FAM or HEX fluorescent adapters. The phenotypic of different alleles were assessed using *t*-test and validated in a panel of 110 advanced breeding lines.

**Figure 3:**
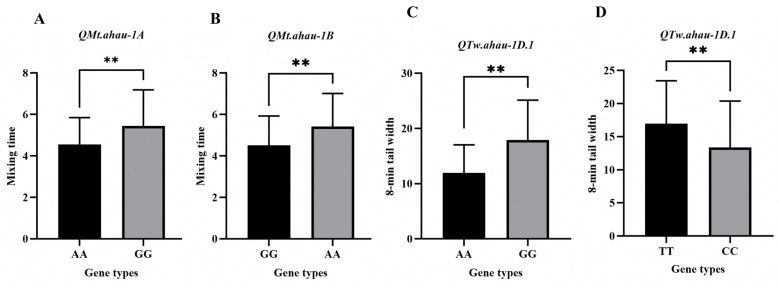
Effect analysis of alleles in the advanced lines with KASP markers. (**A**) Effect analysis of alleles in the advanced lines with *QMt-1A-KASP*. (**B**) Effect analysis of alleles in the advanced lines with *QMt-1B-KASP*. (**C**) Effect analysis of alleles in the advanced lines with *QTw-1D.1-KASP-1*. (**D**) Effect analysis of alleles in the advanced lines with *QTw-1D.1-KASP-2*. ** *p* < 0.01.

These markers were successfully deployed for genotyping in both the original RIL population and the panel of advanced breeding lines. They exhibited clear polymorphism, effectively distinguishing the two genotypes, and the results were fully consistent with the original 55 K SNP array data. Validation in the advanced lines confirmed the phenotypic effects of the favorable alleles (Table S2, Figure 3). Specifically, lines carrying the favorable allele of *QMt-1A-KASP* (*n* = 42) or *QMt-1B-KASP* (*n* = 55) showed a significantly higher average MT of approximately 5.4 min, which was 0.8 min greater than that of lines without the favorable allele. For TW, lines with the favorable allele of *QTw-1D.1-KASP-1* (*n* = 54) and *QTw-1D.1-KASP-2* (*n* = 50) had average values of 17.89% and 16.94%, representing increases of 5.92 and 3.56, respectively, over lines carrying the alternative allele. These results robustly validate the utility of the developed KASP markers for distinguishing phenotypes relevant to wheat quality breeding.

### 2.5. Prediction of Candidate Genes

**Figure 4:**
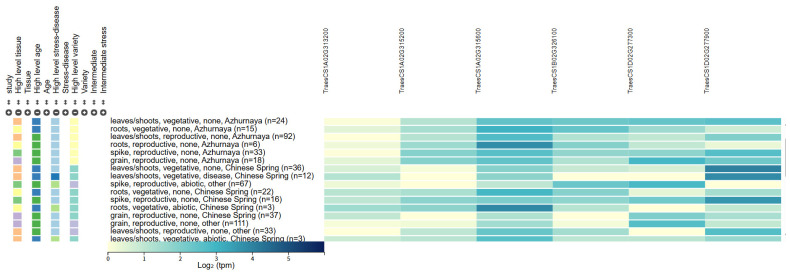
Expression pattern of the candidate genes from a public database (http://wheat-expression.com/) (accessed on 15 February 2026).

Within the physical intervals of the major QTLs *QMt.ahau-1A*, *QMt*/*Pa.ahau-1B*, *QTw.ahau-1D.1*, and *QTw.ahau-1D.2*, a total of 116 high-confidence genes were identified from the Chinese Spring reference genome. Based on their functional annotations and high expression levels in developing grains (as queried from public databases), six genes were prioritized as strong candidates. These encode proteins with diverse functions, including cell-wall modification, transcription regulation, proteolysis, signal transduction, and starch metabolism. Among them, three candidate genes were located on chromosome 1A: *TraesCS1A02G313200* (505.27 Mb), encoding a hydroxyproline-rich glycoprotein family protein; *TraesCS1A02G315200* (506.55 Mb), encoding a CCCH domain zinc finger protein; and *TraesCS1A02G315600* (507.08 Mb), encoding a protease-related family protein. *TraesCS1B02G326100* (551.72 Mb) on chromosome 1B encodes a kinase family protein. Two candidate genes on chromosome 1D, *TraesCS1D02G277300* (374.40 Mb) and *TraesCS1D02G277900* (375.20 Mb), encode phosphoglucan water dikinase and TPR family protein, respectively (Table 5, Figure 4).

## 3. Discussion

The dough-mixing property is a crucial indicator of the rheological changes during dough mixing and is primarily measured using Mixograph. Compared with the Farinograph, Extensograph and Mixolab, the Mixograph offers distinct advantages, including smaller sample requirements and faster testing speed. By recording the resistance during dough mixing, the Mixograph generates a mixing curve, from which key parameters such as mixing time, peak height, peak width, peak area, 8 min tail height, and 8 min tail width, can be derived. These parameters comprehensively reflect the dough’s plasticity [2]. The mixograph parameters show highly significant correlations with core parameters measured by the Farinograph and Extensograph, making them valuable alternative indicators for wheat quality evaluation. In terms of processing quality, parameters like peak area and peak height exhibit high repeatability and are closely related to loaf volume, effectively predicting gluten strength and baking performance [5,6]. The combinations of different mixing parameters can also reflect the suitability of wheat for products such as noodles and steamed bread. As typical quantitative traits controlled by multiple genes, Mixograph parameters are influenced by both genotype and environment. Their QTL mapping is therefore essential for elucidating the genetic mechanisms underlying wheat quality traits.

### 3.1. Comparison with Known QTL Loci

The *QTh.ahau-1A*, associated with 8 min tail height, was stably detected across two environments. It is located at 504.82–508.05 Mb on chromosome 1A and explains an average of 20.38% of the phenotypic variation. This genomic interval is close to the positions of *Glu-1Ax* (*TraesCS1A02G317311*, Refv1.0 chr1A: 508.723–508.726 Mb), and *Glu-1Ay* (*TraesCS1A02G466500LC*, Refv1.0 chr1A: 508.924–508.925 Mb), which encode high-molecular-weight glutenin subunits (HMW-GSs). The importance of this chromosomal region for Mixograph traits is corroborated by prior studies. For instance, Jin et al. [14] conducted an association analysis in 165 cultivars from the Huanghuai wheat region and identified a significant SNP locus for mixing time and 8 min tail width at 506.9 Mb on chromosome 1A, which falls within our mapped interval. Similarly, Kong et al. [15] performed a GWAS on 768 wheat varieties and reported a locus (501.03–511.01 Mb) associated with gluten index and peak height, which partially overlaps with *QTh.ahau-1A*.

*QMt.ahau-1A* was stably detected in two environments, located at 513.62–514.56 Mb on chromosome 1A, with an average phenotypic variation explanation of 15.34% for mixing time. While multiple major QTLs associated with Mixograph properties have been reported on chromosome 1A, such as those for peak height and peak width by James et al. [7] and an SNP marker associated with mixing time reported by Zhang-Biehn et al. [16], the physical position of *QMt.ahau-1A* is distinct from these previously mapped regions. Therefore, *QMt.ahau-1A* likely represents a novel locus.

*QMt.ahau-1B* and *QPa.ahau-1B* were identified as pleiotropic QTLs at 551.72–552.48 Mb on chromosome 1B, explaining an average of 23.62% and 21.72% of the variation for mixing time and peak area, respectively. This region is located approximately 3–4 Mb away from the *Glu-1Bx* and *Glu-1By* genes encoding HMW-GSs. Although a nearby SNP locus affecting mixing time and 8 min tail width were reported at 553.6 Mb [14], the interval identified here does not overlap with it nor with other previously reported QTLs for similar traits on chromosome 1B, such as *QMPT.sdau-1B* (180.31 Mb) and *QMPT1B.1-3* (152.07 Mb) [17]. Therefore, *QMt*/*QPa.ahau-1B* is proposed to be a novel genetic locus.

The *QTw.ahau-1D.2* locus for 8 min tail width was mapped to 410.07–413.06 Mb on chromosome 1D, a region that overlaps with the well-characterized Glu-D1 locus encoding high-molecular-weight glutenin subunits (HMW-GS). Previous studies have consistently reported significant effects of the Glu-D1 region on the mixing time and peak-related traits [13,18]. Furthermore, a genome-wide association analysis identified an SNP locus for the mixing time and tail width at 407.9–416.5 Mb on chromosome 1D, a region that encompasses *Glu-D1x* and *Glu-D1y* [14]. In contrast, *QTw.ahau-1D.1* (374.05–375.94 Mb) resides in a distinct genomic interval and explains an average of 9.89% of the variation for 8 min tail width. To our knowledge, this region has not been previously linked to Mixograph properties, suggesting that *QTw.ahau-1D.1* may represent a novel QTL.

All stable QTLs identified in this study are located on chromosomes 1A, 1B, and 1D. These chromosomes have been widely recognized as core genomic regions harboring major loci-controlling Mixograph properties [8,10]. On chromosome 1A, the Glu-A1 locus has been repeatedly associated with parameters such as peak time and 8 min tail height [15]. For chromosome 1B, multiple functional loci, including those within or adjacent to the Glu-B1 region, have been reported to influence mixing time, peak area, and other traits [15,17,18]. Notably, some of these loci exhibit pleiotropic effects and may form gene clusters [18]. Chromosome 1D, particularly the Glu-D1 locus, is a major focus of research due to its substantial and pleiotropic effects on numerous dough quality traits, including Mixograph parameters and Farinograph stability time [12,15,18]. The superior effect of the Glu-D1 5 + 10 subunit over the 2 + 12 subunit is well-established [17]. Furthermore, large-scale QTL and GWAS studies have consistently identified numerous significant loci on these chromosomes, underscoring their central role in determining dough-mixing behavior [8,15,18]. The co-localization of the QTLs mapped in this study with these known critical regions reinforces the importance of group 1 chromosomes in the genetic control of wheat Mixograph properties. Future work should include a direct genotyping of the HMW-GS composition to definitively confirm the relationship between these known major loci and our identified QTL intervals. This would provide a clearer understanding of the genetic basis underlying the Mixograph properties.

*QMt.ahau-1A*, *QMt*/*QPa.ahau-1B*, *QTw.ahau-1D.1* were potentially novel loci. We propose that they may represent additional, perhaps more subtle, genetic factors such as regulatory elements or genes involved in other pathways like starch metabolism or protein processing, which collectively contribute to the complex genetic architecture of Mixograph properties.

### 3.2. Development of Molecular Markers for Major Loci, QMt.ahau-1A, QMt.ahau-1B, and QTw.ahau-1D.1

Conventional breeding methods face limitations in selection efficiency for quality traits, primarily due to the technical difficulty, lengthy cycle, and cumbersome nature of phenotyping quality traits from field-grown materials [19]. The emergence of KASP technology provides a promising platform for MAS, owing to its economy, flexibility, and accuracy. In this study, four functional KASP markers (*QMt-1A-KASP*, *QMt-1B-KASP*, *QTw-1D.1-KASP-1*, *QTw-1D.1-KASP-2*) were successfully developed based on tightly linked SNP loci for the target loci. Validated in 110 advanced breeding lines confirmed that these markers can efficiently and accurately identify alleles associated with Mixograph properties. This study successfully translates molecular markers into practical breeding tools, thus enhancing selection efficiency and predictability. However, the general applicability, stability, and predictive accuracy of these markers across genetically diverse wheat materials require further systematic validation. Therefore, multi-population and multi-environment trials are essential to fully evaluate their practical utility in marker-assisted selection (MAS).

### 3.3. Candidate Genes

Based on the Chinese Spring wheat genome, six candidate genes were identified within the target loci. Their functional annotations suggest direct or indirect roles in determining Mixograph traits. On chromosome 1A, three genes were prioritized. *TraesCS1A02G313200* encodes a hydroxyproline-rich glycoprotein (HRGP), a structural cell-wall protein implicated in tissue stability and stress response, potentially influencing endosperm cell-wall properties and processing quality [20,21]. *TraesCS1A02G315200* encodes a CCCH-type zinc finger transcription factor involved in growth and stress adaptation, which may regulate storage protein expression to balance dough strength and extensibility [22,23,24]. *TraesCS1A02G315600* encodes a protease-related family protein; such proteins, fundamental to protein turnover, could alter dough rheology by modulating gluten hydrolysis [25]. On chromosome 1B, *TraesCS1B02G326100* encodes a protein kinase. While crucial for signaling, few wheat kinases are functionally characterized [26]; it is hypothesized to influence grain development or storage protein synthesis. On chromosome 1D, the analysis focused on the *QTw.ahau-1D.1* locus, given the well-established role of HMW-GS genes nearby. *TraesCS1D02G277300* encodes phosphoglucan water dikinase (PWD), a key starch metabolic enzyme. Its downregulation reduces starch phosphate and increases grain size and α-amylase activity [27], suggesting that PWD coordinates starch synthesis and degradation to affect mixing behavior. *TraesCS1D02G277900* encodes a TPR superfamily protein that mediates protein–protein interactions [28]; it may link gluten synthesis with starch metabolism to regulate Mixograph properties.

## 4. Materials and Methods

### 4.1. Plant Materials and Field Experimental Design

The experimental material consisted of an F_6_ RIL population derived from a cross between Yangxiaomai and Zhongyou 9507, which included 227 lines. Additionally, 110 advanced breeding lines were used as the validation materials for the KASP markers. All materials were provided by Professor Xianchun Xia from Chinese Academy of Agricultural Sciences.

The RIL population was evaluated in multiple environments: in Suixi, Anhui (E1) during the 2020–2021 growing season, and in Hefei, Anhui (E2), as well as Suixi, Anhui (E3), during the 2021–2022 growing season. Each line was sown in two rows of 2 m (m) rows with a row spacing of 25 cm, using 30 seeds per row, and it was arranged in three replications. The 110 advanced breeding lines were planted in Xinxiang, Henan, across the 2020–2021 and 2021–2022 growing seasons, following a completely randomized block design with three replications. Each plot consisted of six rows, each 4.5 m in length with a row spacing of 20 cm. All field management practices were carried out in accordance with local standard protocols.

### 4.2. Phenotypic Measurement

After harvesting, wheat grains were air-dried and milled into flour using a Brabender Junior experimental mill (Brabender Inc., Duisberg, Germany), yielding a flour extraction rate of approximately 60%. For each environment, grain samples from the three field replications were bulked prior to milling to obtain a representative sample. The milling was performed once per entry per environment. Mixograph parameters including mixing time, peak area, 8 min tail height, and 8 min tail width, which were measured using a 10-g Mixograph (National Manufacturing, TMCO Division, Lincoln, NE, USA), following the AACC 08-01 method [12].

### 4.3. Statistical Analysis of Phenotypic Data

Best linear unbiased prediction (BLUP) values were calculated with the lme4 package in R software (https://www.r-project.org/) (accessed on 25 June 2024). Both the environment-specific means and the BLUP values of phenotypic data were subsequently used for the phenotypic and genetic analyses. The descriptive statistics and gene effect analyses were performed using GraphPad Prism v10. The analysis of variance (ANOVA), correlation analysis, and the calculation of broad-sense heritability (*H*^2^) were conducted using IciMapping v4.2. Finally, frequency distribution diagrams for each Mixograph parameter across different environments were generated using Origin 2024.

### 4.4. Construction of Genetic Linkage Map and QTL Mapping

A previously published high-density genetic linkage map constructed with 55 K SNP array data from the RIL population and parental lines [29] was employed for the QTL analysis. QTL mapping was conducted using the inclusive composite interval mapping (ICIM) method in the IciMapping v4.2 software, with a significance LOD threshold set at 2.5. Putative QTLs with intervals less than 10 cM or sharing flanking markers were considered to represent the same locus. A major QTL was defined as one detected in at least two environments and explaining >10% of the phenotypic variance. Positive additive effects indicate that the trait increase originated from Zhongyou 9507, whereas negative effects corresponded to alleles from Yangxiaomai. QTLs were named according to the convention: “Q” + trait abbreviation + “.ahau” (Anhui Agricultural University) + chromosome.

### 4.5. Development and Validation of KASP Markers

Based on closely linked markers and the confidence intervals of the major QTLs, the corresponding flanking sequences were extracted from the WheatOmics database (http://202.194.139.32/) (accessed on 14 June 2024). These sequences were subsequently submitted to the Polymarker website (http://www.polymarker.info/) (accessed on 25 June 2024) for the design of KASP assays. Four sets of KASP primers, along with their common primers, were successfully designed and synthesized by Sangon Biotech Co., Ltd. (Shanghai, China). (https://store.sangon.com/) (accessed on 1 July 2024). The PCR reaction was carried out in a 5 µL system containing: 2.0 µL of 2×KASP Master Mix, 0.0336 µL of the primer mix (a combination of two allele-specific forward primers and one common reverse primer at a final total concentration of 50 µM, mixed at a molar ratio of 2:2:5), and 2 µL template DNA (50 ng/µL). Amplification was performed on a water bath PCR instrument (LGC Ltd., Hoddesdon, UK) with the following thermal profile: an initial denaturation at 94 °C for 15 min; 10 touchdown cycles of 94 °C for 20 s and 65–57 °C for 60 s (decreasing by 0.8 °C per cycle); followed by 35 cycles of 94 °C for 20 s and 57 °C for 60 s; and a final storage at 4 °C in the dark.

Genotyping of the 110 advanced breeding lines was performed using a 384-well plate format on a PHEstar instrument (BMG Labtech GmbH, Ortenberg, Germany). Following the fluorescence scan, the genotyping data were processed and analyzed using the KLUSTER Caller v4.1.2 (LGC, Hoddesdon, UK) to visualize the allelic clusters and thereby validate the effectiveness of the markers and their associated QTLs.

### 4.6. Screening of Candidate Genes

To delineate the physical regions harboring the QTLs, the sequences of tightly linked molecular markers were aligned via BLAST (Chinese Spring gene, Ref Seq v1.0) to determine their genomic positions. Subsequently, high-confidence annotated genes within these target intervals were retrieved from the IWGSC RefSeq v1.1 genome assembly (https://wheat-urgi.versailles.inra.fr/) (accessed on 10 June 2024). For the expression profiles across different tissues and development stages were further investigated using the publicly available hexaploid wheat transcriptom database on the wheat-expression website (https://www.wheat-expression.com/) (accessed on 15 February 2026).

## 5. Conclusions

This study successfully identified six stable genetic loci associated with key Mixograph parameters, including mixing time, peak area, 8 min tail width, and 8 min tail height in an F_6_ RIL population derived from a cross between Yangxiaomai and Zhongyou 9507. These loci individually accounted for phenotypic variation ranging from 4.09–28.70%. Among them, *QTh.ahau-1A*, *QMt*/*QPa.ahau-1B*, and *QTw.ahau-1D.1* represent potentially novel loci, thereby enhancing the genetic resources avaliable for wheat quality breeding. We further developed and preliminarily validated four practical KASP markers for marker-assisted selection. Six candidate genes were identified from the QTL regions. Future work should involve fine-mapping, multi-background marker validation, and functional studies. These findings provide essential genetic tools for enhancing Mixograph properties in wheat breeding.

## Figures and Tables

**Table 1:** Phenotypic distribution of Mixograph parameters in wheat parents and their RIL populations.

Trait	Environment	Parents		RILs			
		Zhongyou 9507	Yangxiaomai	Minimum	Maximum	Mean	C.V.
MT	E1	4.4	2.1	1.3	5.2	2.6	0.28
	E2	3.9	1.6	1.3	4.9	2.6	0.24
	E3	3.1	1.8	1.3	4.4	2.4	0.24
	E4	3.8	1.8	1.6	4.4	2.5	0.2
PA	E1	177.04	84.04	53.05	247.81	112.68	0.28
	E2	180.49	75.11	58.96	239.54	117.03	0.25
	E3	139.03	79.58	56.12	205.98	115.45	0.24
	E4	165.52	79.58	66.09	201.12	115.05	0.19
TH	E1	43.63	41.12	37.93	51.81	43.91	0.05
	E2	49.4	44.23	37.32	55.57	44.92	0.06
	E3	48.09	42.67	36.22	57.04	46.66	0.08
	E4	47.04	42.67	39.56	50.12	45.16	0.04
TW	E1	10.34	3.91	2.77	14.47	6.9	0.37
	E2	5.89	5.67	2.59	12.53	5.74	0.33
	E3	6.71	4.79	2.59	17.07	6.28	0.39
	E4	7.65	4.79	4.17	10.32	6.3	0.18

MT: mixing time; PA: peak area; TH: 8 min tail height; TW: 8 min tail width. E1, E2, E3, and E4 indicate Suixi 2021, Suixi 2022, Hefei 2022, and the best linear unbiased prediction (BLUP), respectively. C.V.: coefficient of variation.

**Table 2:** ANOVA and *H*^2^ of Mixograph parameters in RIL population.

Trait	Mean Square			F-Value			*H* ^2^
	Genotype	Environment	G × E	Genotype	Environment	G × E	
MT	3.02	7.18	0.34	174.58 **	414.23 **	19.83 **	0.90
PA	6169.98	3304.69	885.31	24,089.16 **	12,902.33 **	3456.49 **	0.87
TH	46.98	1320.2	14.11	411.48 **	11,562.92 **	123.59 **	0.77
TW	26.37	234.67	9.47	1916.38 **	17,051.77 **	687.97 **	0.74

** *p* < 0.01. MT: mixing time; PA: peak area; TH: 8 min tail height; TW: 8 min tail width. *H*^2^: broad-sense heritability.

**Table 3:** Analysis of stable QTLs for Mixograph parameters in RIL population.

Trait	QTL	Flanking Markers	Physical Interval (Mb)	LOD Value	PVE (%)	Additive Effect	Environment
MT	*QMt.ahau-1A*	*AX-110911310*-*AX-108914408*	513.62–514.56	4.59	15.34	0.23	E2/E3
	*QMt.ahau-1B*	*AX-111503025*-*AX-110519906*	551.72–552.48	21.23	23.62	0.39	E1/E2/E4
TW	*QTw.ahau-1D.1*	*AX-109827648*-*AX-110069135*	374.05–375.94	5.05	9.89	−0.54	E1/E4
	*QTw.ahau-1D.2*	*AX-109079356*-*AX-111804778*	410.07–413.66	6.28	10.32	0.84	E1/E3/E4
PA	*QPa.ahau-1B*	*AX-111503025*-*AX-110519906*	551.72–552.48	23.2	21.72	14.18	E1/E4
TH	*QTh.ahau-1A*	*AX-109055961*-*AX-111496677*	504.82–508.05	13.08	20.38	−1.11	E2/E4

MT: mixing time; PA: peak area; TH: 8 min tail height; TW: 8 min tail width. The physical interval was referenced against the IWGSC v1.0.PVE: phenotypic variation explanation. E1, E2, E3, and E4 indicte Suixi 2021, Suixi 2022, Hefei 2022, and the best linear unbiased prediction (BLUP), respectively.

**Table 4:** Developed KASP primer sequences based on major QTLs.

KASP Marker	SNP Marker	FAM-Compatible Primers (5′-3′)	HEX-Compatible Primers (5′-3′)	Common Primers (5′-3′)
*QMt-1A-KASP*	*AX-110911310*-*AX-108914408*	gcatgtcatcagcactgtgA	gcatgtcatcagcactgtgG	cctttctcacataTggcgcC
*QMt-1B-KASP*	*AX-111503025*-*AX-110519906*	ccacaactttctgcctagctA	ccacaactttctgcctagctG	ttatgcggtCgaggatccG
*QTw-1D.1-KASP-1*	*AX-109827648*-*AX-110069135*	tcaacacTaaatgctccaaaaacaG	tcaacacTaaatgctccaaaaacaA	gcttgctaacctggaagatccG
*QTw-1D.1-KASP-2*	*AX-109827648*-*AX-110069135*	cttCgtccacgtccAttagT	cttCgtccacgtccAttagC	acacaagagcatgtaataccgG

**Table 5:** Candidate genes for Mixograph-related traits.

Candidate Gene	Chromosome	Position	Annotation
		(Mb)	
*TraesCS1A02G313200*	1A	505.27	Hydroxyproline-rich glycoprotein family protein
*TraesCS1A02G315200*	1A	506.55	Zinc finger CCCH domain protein
*TraesCS1A02G315600*	1A	507.08	Protease-related family protein
*TraesCS1B02G326100*	1B	551.72	Kinase family protein
*TraesCS1D02G277300*	1D	374.40	Phosphoglucan, water dikinase, chloroplastic
*TraesCS1D02G277900*	1D	375.20	Tetratricopeptide repeat (TPR)-like superfamily protein

## Data Availability

Data are contained within the article and Supplementary Materials.

## Supplementary Materials

The following supporting information can be downloaded at: https://www.mdpi.com/article/10.3390/plants15071016/s1, Figure S1: Genetic Linkage map QTLs co-localized for the Mixograph parameters in the RIL population; Table S1: Mapping of QTLs for Mixograph parameters in the RIL population; Table S2: Allelic segregation and effect analysis of KASP markers in advanced lines.
