# Supplementary figures and images for: Genome-Wide Linkage Mapping for Mixograph Properties in Common Wheat
Source: Plants (Basel). 2026 Mar 26;15(7):1016. doi: 10.3390/plants15071016 (PMC13074796; doi:10.3390/plants15071016)

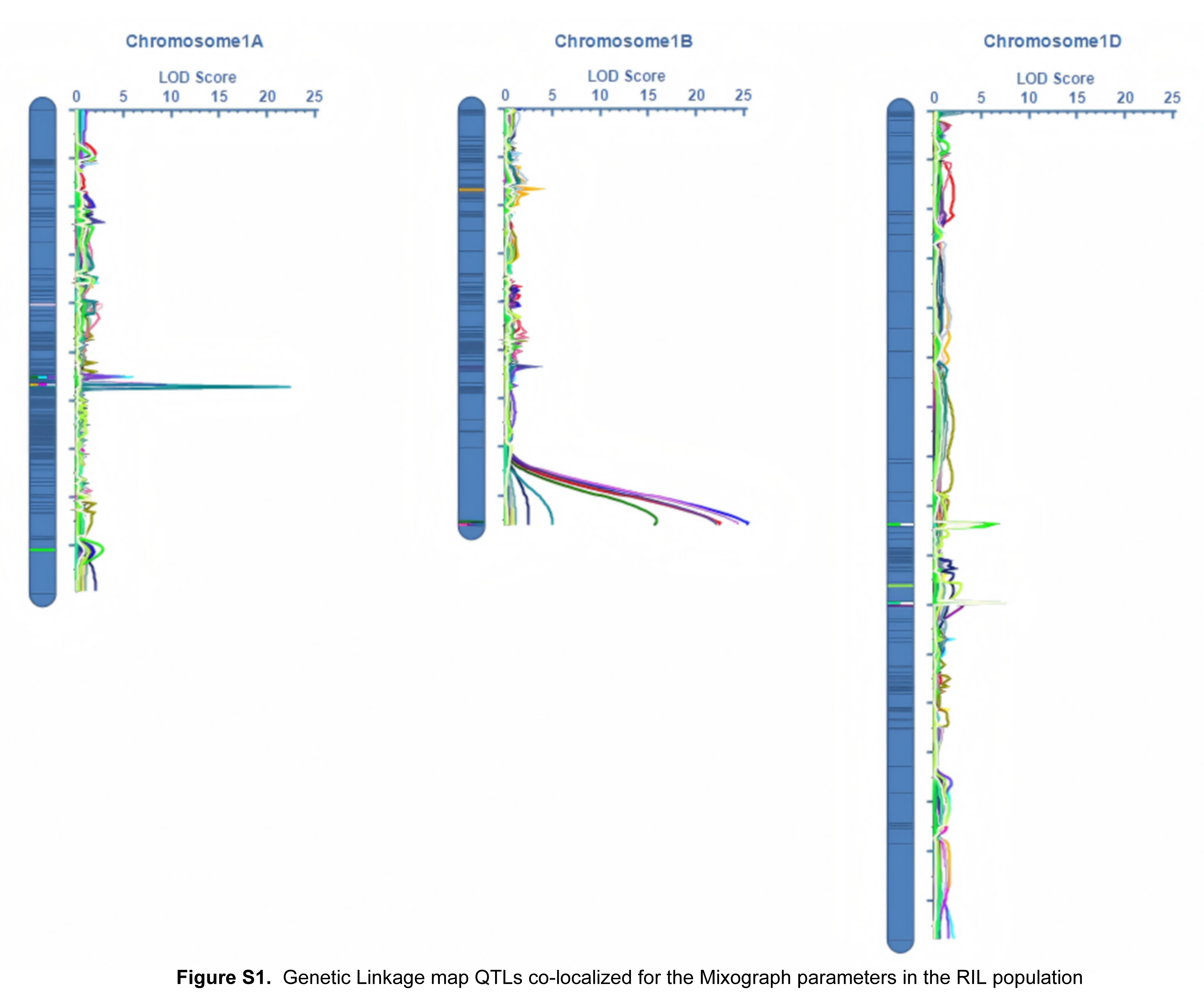

Supplement: Supplementary file 1 [file plants-15-01016-s001.zip › Supplementary Figure S1.png]
